# St. Mary's Hospital, Paddington

**Published:** 1902-03-29

**Authors:** 


### ST. MARY'S HOSPITAL, PADDINGTON.

THE YEAR'S WORK.

The subject of the Clarence Wing has been occupying the minds of the Board of Management during the past year, and the report opens with the statement that the £50,000 required to complete the building have at last been obtained.

A £25,000 Prize.

The late Mr. Siegfried Rudolph Zunz left the sum of £25,000 to his executors to be bequeathed, at their discretion, to one of the metropolitan hospitals "for the construction, maintenance, and endowment, or any of those purposes, of a ward to be called in perpetuity the Annie Zunz Ward." After very careful consideration of the many applications received, the hospital selected was St. Mary's, and in gratitude for this the executors, Messrs. Henry Gardner, C. L. Budd, and Samuel Baer, have been elected honorary life governors of the hospital, and the first named gentleman has been made a member of the Board of Management. The condition attached to the award was that the further £25,000 required for completing the Clarence Wing (which had been stopped for want of funds) must be raised within six months. This condition, viewed by some of the board with disfavour, has been fulfilled, the executors' cheque has been paid, and the sum temporarily invested in Consols.

How was the Money Raised?

The subscription list was headed by the president of the hospital, the Prince of Wales, and this fact, together with the influence and liberality of the treasurer, Mr. Alfred de Rothschild, no doubt accounts for the rapid success attending the appeal. Mr. Rothschild is responsible for two anonymous sums amounting to £4,500, and Messrs. N. M. Rothschild and Sons gave £3,000; four other donations of £1,000 helped to swell the total amount, and smaller sums, including £250 from the Great Western Railway Company, and £292 1s. 10d., the proceeds of balls arranged by Mrs. Field, are included among large sums. The Salters' Company gave £105, and the Tylers and Bricklayers £31 10s. Directly the sum was raised the King was informed of the fact, since his Majesty, having laid the foundation-stone of the Clarence Wing, was known to take great interest in the building. The King replied that he was very pleased to learn that the work was to be resumed, and a similar message came by cable from the Prince of Wales, then at Niagara. As soon as possible steps were taken to proceed with the building, which will now go forward, it is hoped, without interruption. In the interval some important alterations have been decided upon, which necessitate considerable revision of the plans, and, a more serious result, some delay; the cost of labour also has risen, making a probable difference of 10 per cent. on the outlay. This, with outlay on fittings and furniture and some considerable structural alterations in the interior of the old building entailed by the enlargement scheme, will necessitate the raising of about £10,000 more.

Routine Work: Finance.

Apart from the excitement of raising a large sum of money for a special purpose, the regular work of the year has been fairly satisfactory, though the ordinary income was less by £1,114 than in 1900. This was partly caused by a decline in dividends, owing to the sale of stock to meet the deficiency at the end of 1900; and partly by the special efforts referred to above, which had the effect of lessening the annual contributions. The general maintenance fund, however, is better off by £3,762 than in 1900, legacies being higher by £1,926, and endowment donations and legacies directed to be invested by £2,950. The total income available to meet expenditure was £23,155 18s. 9d.; the total expenditure £28,808 12s. 6d. Expenditure was heavier in 1901 than even in 1900, the increase being chiefly under the heads of surgery and dispensary and domestic expenses; the first on account of the increased expense of modern methods, and the last mainly on account of coal and gas, gas alone having gone up at the rate of 17 per cent. The general expenditure has also been increased by the necessity of lighting, cleaning, warming, and paying rates for the basement of the new wing, so that it would not be fair this year to take an average of the cost per bed. Four beds and one cot have been endowed, bringing the number up to 35, 17 for men, 12 for women, and six for children. All the endowments have been made since 1893, with one exception.

Routine Work: Patients.

The number of patients relieved is the highest on record, viz, 47,493: in-patients, 3,812; out-patients, 23,029; casualties, 18,829; maternity, 1,823. The average number resident was 250, the mean residence of each being 24·1 days. The rate of mortality was 8·69 per cent. The system of enquiring into the circumstances of the out-patients, with a view to the prevention of abuse, has been continued; 174 of the cases calling for special investigation were found to be unsuitable, a number equal to ·78 per cent. on all patients. Notwithstanding the adoption of this system, the out-patients were more numerous by 1,778 than in 1900. Many of the patients are very poor, and the secretary has constantly to discharge them at a time when their physical powers of existence are at the lowest ebb, in a half-clad condition. Gifts of cast-off clothing, especially of boots and shoes, are earnestly solicited, the many gifts received being still inadequate for this purpose. The Council of King Edward's Hospital Fund made a thorough inspection of the hospital last June, and expressed themselves as pleased with what they saw. Of their grant of £1,200, £1,000 was made with the expressed object of "encouraging the committee to continue the work of meeting the deficiency between the income and expenditure."

